# Congenital Craniofacial Plexiform Neurofibroma in Neurofibromatosis Type 1

**DOI:** 10.3390/diagnostics11020218

**Published:** 2021-02-02

**Authors:** Antonella Cacchione, Alessia Carboni, Mariachiara Lodi, Rita De Vito, Andrea Carai, Antonio Marrazzo, Marina Macchiaiolo, Ioan Paul Voicu, Angela Mastronuzzi, Giovanna Stefania Colafati

**Affiliations:** 1 Department of Paediatric Haematology/Oncology, Cell and Gene Therapy, Bambino Gesù Children’s Hospital, IRCCS, 00165 Rome, Italy; mariachiara.lodi@opbg.net (M.L.); angela.mastronuzzi@opbg.net (A.M.); 2 Oncological Neuroradiology Unit, Department of Imaging, Bambino Gesù Children’s Hospital, IRCCS, 00165 Rome, Italy; alessia.carboni@opbg.net (A.C.); antonio.marrazzo@opbg.net (A.M.); gstefania.colafati@opbg.net (G.S.C.); 3 Histopathology Unit, Bambino Gesù Children’s Hospital, 00165 Rome, Italy; rita.devito@opbg.net; 4 Neurosurgery Unit, Department of Neuroscience and Neurorehabilitation, Bambino Gesù Children’s Hospital, IRCCS, 00165 Rome, Italy; andrea.carai@opbg.net; 5 Rare Diseases and Medical Genetic Unit, Bambino Gesù Children’s Hospital, 00165 Rome, Italy; marina.macchiaiolo@opbg.net; 6 Radiodiagnostics Unit, Giuseppe Mazzini Hospital, 64100 Teramo, Italy; ioanpaul.voicu@aslteramo.it

**Keywords:** plexiform neurofibroma, neurofibromatosis type I, MRI, target sign

## Abstract

We present a case demonstrating the performance of different radiographical imaging modalities in the diagnostic work-up of a patient with neurofibromatosis type 1 (NF1) and plexiform neurofibroma (PN). The newborn boy showed an expansive-infiltrative cervical and facial mass presented with macrocrania, craniofacial disfigurement, exophthalmos and glaucoma. A computer tomography (CT) and a magnetic resonance imaging (MRI) were performed. The CT was fundamental to evaluate the bone dysmorphisms and the MRI was crucial to estimate the mass extension. The biopsy of the lesion confirmed the suspicion of PN, thus allowing the diagnosis of NF1. PN is a variant of neurofibromas, a peripheral nerves sheath tumor typically associated with NF1. Even through currently available improved detection techniques, NF1 diagnosis at birth remains a challenge due to a lack of pathognomonic signs; therefore congenital PN are recognized in 20% of cases. This case highlights the importance of using different radiological methods both for the correct diagnosis and the follow-up of the patient with PN. Thanks to MRI evaluation, it was possible to identify earlier the progressive increasing size of the PN and the possible life threatening evolution in order to perform a tracheostomy to avoid airways compression.

The patient was born at the thirty-eighth week of gestational age by caesarean section following the finding of cerebral ventriculomegaly on last trimester ultrasound. At birth, the child had the following anthropometric parameters: weight 3090 g (34 °p), length 51 cm (77 °p), and head circumference 37.5 cm (100 °p). He presented numerous dysmorphisms due to the presence of a right abnormal facial and laterocervical mass, including macrocrania and craniofacial asymmetry with greater development of the right half-face, exophthalmos of the right eye with glaucoma.

The axial T2-weighted (T2W) sequences on MRI showed an ill-defined giant cervical and facial mass of the right side (Figure 1a), infiltrating the deep spaces of the neck, showing infra-temporal fossa and intra-orbital extension (Figure 1b) and parotid gland infiltration (Figure 1a). The intracranial extension into the skull base followed the course of the right trigeminal nerve (Figure 1c, white arrows). The mass involved the deep regional soft tissues and invaded the surrounding structures, determining a proptosis of the right eye due to right side orbital enlargement. The solid part of the extensive mass was characterized by intermediate to high signal intensity on T2 and intermediate to low signal intensity on non-enhanced T1w with enhancement after gadolinium injection (Figure 1a–c). The optic nerve and extra-ocular muscles were involved by the mass. The typical MRI target-sign, namely central hypointensity with peripheral hyperintensity on T2 weighted images, was suggestive for the diagnosis of plexiform neurofibroma (PN).

The histologic examination after mass biopsy revealed diffuse nerve fascicles distension caused by a proliferation of spindle-shaped cells embedded in myxoid matrix (hematoxylin-eosin (EE), 2.5×) consistent with plexiform neurofibroma (PN) (Figure 1d).

PNs arise from multiple nerve and are commonly associated with neurofibromatosis type I (NF1). Although histologically benign, PN are associated with significant morbidity and mortality, both because surgical treatment is complex due to their usually very extensive and compressing ever infiltrative nature and because they are highly resistant to conventional chemotherapy. They are usually congenital, but they may instead present during the first year as a subtle soft-tissue enlargement or a large patch of cutaneous hyperpigmentation. They occur in at least 50% of patients with neurofibromatosis type I (NF1). Many are asymptomatic and the exact timing of growth of plexiform tumors is unpredictable. Boulanger JM et al. [1] clear show how the symptoms depending on the location of the tumors. Tumors of the head, neck and face are most common followed by disfigurement and lesions of the spine, extremities and abdomen. They often arise from the dorsal spinal roots, nerve plexi, large peripheral or sympathetic chains. Plexiform tumors may be discrete, homogenous and well circumscribed or diffuse, heterogeneous and infiltrative. They may involve superficial skin or be entirely internal.

NF1 is one of the most frequent hereditary neurocutaneous disorders with a birth incidence of about 1:2500. NF1 is caused by heterozygous germ-line mutations in the tumor suppressor gene NF1, located on chromosome 17q11.2. NF1 encodes a protein called neurofibromin, which functions, in part, as a negative regulator of the Ras proto-oncogene, a key signaling molecule of cell growth. NF1 has a high inter and intra familiar clinical heterogeneity [2]. The diagnosis of NF1 is established in a proband who meets the diagnostic criteria for NF1 developed by the National Institutes of Health (NIH). At least two of the following seven diagnostic criteria must be met to diagnose NF1 [2,3]:

- Six or more café au lait macules >5 mm in greatest diameter in prepubertal individuals and >15 mm in greatest diameter in postpubertal individuals
- Two or more neurofibromas of any type or one plexiform neurofibroma
- Freckling in the axillary or inguinal regions
- Optic glioma
- Two or more Lisch nodules (iris hamartomas)
- A distinctive osseous lesion such as sphenoid dysplasia or tibial pseudarthrosis
- A first-degree relative (parent, sib, or offspring) with NF1 as defined by the above criteria

The diagnosis is in some cases difficult since many of the features are age-dependent.

About 50% of individuals with neurofibromatosis type 1 have no family history of the disease and the disease is due to de novo mutations. In our case a specific mutation of the NF1 gene was detected (*c.1318C > T*). Further investigations, only after the newborn diagnosis of NF1, revealed that the child’s mother had the clinical stigmata of NF1 confirmed by the presence of the same constitutional mutation. She had also previously undergone surgery for a cutaneous neurofibroma, without receiving further genetic counseling.

The presence of at least one PN is one of the diagnostic criteria. In clinical practice, the first manifestation of the disease can be a congenital PN.

In our patient, no other signs of neurofibromatosis were detected by clinical examination, as typically happens in newborn children, based on our clinical practice.

Since at first axial T2-weighted MRI images (T2WI) performed at birth, the characteristic sign of the neuronal origin of the lesion, defined as “target sign” (Figure 2a), was evident. The typical T2w appearance of the “target sign” is a rim of high signal peripherally and low signal centrally that corresponds pathologically to central fibrocollagenous tissue and peripheral predominantly myxoid tissue [1,2]. These findings were confirmed at MRI performed during the follow up (Figure 2b). Neurofibroma appears generally hypointense on T1w with a mild enhancement after contrast injection. Matsumine et al. show that the T2w sequence is helpful in differentiating neurofibromas from histologically malignant peripheral nerve sheath tumors [4]. Moreover conventional MRI features, such as regular or irregular margins and T1W post-contrast heterogeneity, are helpful for differentiating benign from malignant peripheral nerve sheath tumors and can be very challenging using conventional MR imaging [5,6]. The diffusion weighted images (DWI) sequence and apparent diffusion coefficient (ADC) map, into a conventional MRI examination, has a potential role for distinguishing benign and malignant neurofibromas and may impact the clinical care of patients with these tumors [7,8].

In order to define the lesion and the subsequent facial alterations more precisely, CT scan was performed at birth and it underlined bony defects, such as thickened and sclerotic bone and dysplastic change, involving the sphenoidal bone, the orbital cavity and the adjacent right skull base (Figure 2e). The right orbital cavity was enlarged, and the right maxilla was dysplastic. The CT surface shaded display (SSD) reconstruction showed the cosmetic impairment of right face, proptosis of the right eye, and the right ear was lower than normal in location (Figure 2d). On CT, the neurofibroma appears as a hypodense mass with intralesional vessels and a heterogeneous and variable enhancement after contrast agent injection. The aspect is usually of a non-specific infiltrative lesion. MRI is the gold standard imaging technique for the diagnosis and the local staging of the neurofibromas by providing useful and precise data extension, location and regional anatomical structures involvement. However, particularly on superficial locations, the absence of a target-like appearance does not rule out the diagnosis. In our case, the evidence of the target sign on the lesion extended on the mid skull base of the right side suggested for this diagnosis.

The T2 target sign in our case was more visible at the follow-up (Figure 2a,b). In our case, the patient’s PN showed a progressive volumetric increase in PN, thus allowing early identification of the high risk of airway obstruction. It was therefore possible to schedule a surgical tracheostomy (Figure 2e).

PNs are typically congenital, but they show their clinical evidence between 2 and 5 years [3]. Differential diagnosis based on imaging includes lymphatic or venous malformation, hemangioma, traumatic or inflammatory lesion. PNs can involve any peripheral nerve and are generally extensive with undefined margins [2]. They occur frequently in the head, neck, face, and larynx, especially the cranial and upper cervical nerves [2,9]. The cranial nerves commonly involved are the fifth, ninth, and tenth. They are associated with major nerve trunks or plexi, may appear as a large, disfiguring mass [9]. They are usually solitary lesions; nevertheless, around 5–10% occur as multiple lesions. They can progress to malignant peripheral nerve sheath tumor with an incidence of nearly 5–6% [3]. They may have a superficial or deep location. PNs usually present with ill-defined margins and signs of increased vascularity.

They are highly resistant to conventional chemotherapy; therefore, the management of PNs in the pediatric patient consists of serial follow up and observation. Surgery still remains the main stay of treatment but is reserved for the most severe of cases [10]. In case of extensive surgical resection, preoperative imaging should be performed for the surgical planning. In this scenario, both CT and MRI provide complementary information to the surgeon. MRI is helpful in assessing the PN margins and the relationship with surrounding structures. Conversely, CT scan is more suitable to reveal bone involvement and relationships between the PN and the bony structures.

Differential diagnosis based on imaging includes lymphatic or venous malformation, hemangioma, traumatic or inflammatory lesions.

In conclusion, NF1 is one of the most frequent hereditary neurocutaneous disorders. In clinical practice, the first manifestation of the disease can be a congenital PN, especially in newborns, as in our case. MRI is immensely helpful to guide the correct diagnostic hypothesis and to weigh the differential diagnoses and eventual malignant transformation. Radiologists must be aware of typical MRI appearance of PN. This case highlights the importance of using different radiological methods (CT and MRI) for the correct diagnosis and the follow-up of the patient with PN. However, CT scan should be used sparingly in patients affected by NF1, and may provide valuable information in cases of extensive PN resection. Thanks to MRI evaluation, it was possible to identify earlier the progressive increasing size of the PN and the possible life-threatening evolution in order to perform a tracheostomy to avoid airways compression.

In case of extensive surgical resection, preoperative imaging should be performed or the surgical planning. In this scenario, both CT and MRI provide complementary information to the surgeon. MRI is helpful in assessing the PN margins and the relationship with surrounding structures. Conversely, CT scan is more suitable to reveal bone involvement and relationships between the PN and the bony structures.

Different radiological techniques are fundamental for the management of PNs. They allow detection of the correct diagnosis and the eventual malignant transformation. MRI and TC evaluations guide also the correct follow-up, intervening only in those cases selections that really require surgery.

This case highlights the importance of using different radiological methods (CT and MRI) for the correct diagnosis and the follow-up of the patient with PN. Thanks to MRI evaluation, it was possible to identify earlier the progressive increasing size of the PN and the possible life-threatening evolution in order to perform a tracheostomy to avoid airways compression.

## Figures and Tables

**Figure 1:**
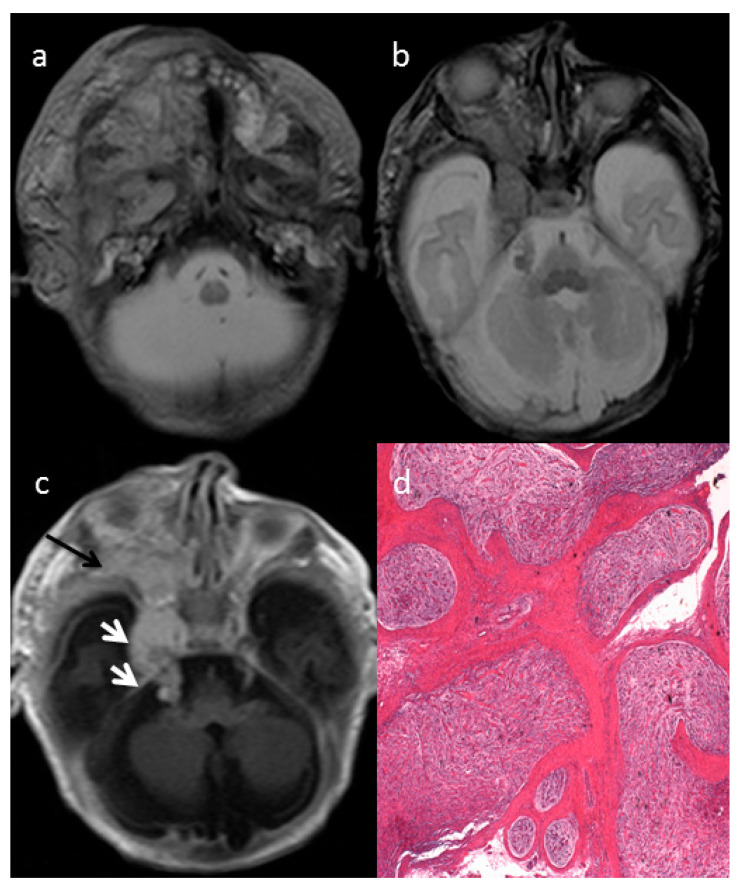
The axial T2-w (**a**,**b**) and T1-w (**c**) of the MRI showed an ill-defined giant cervical and facial mass of the right side on the neck deep spaces with infra-temporal fossa and intra-orbital extension and parotid gland infiltration. The intracranial extension followed the course of the right trigeminal nerve ((**c**) white arrows). The mass involved the deep regional soft tissues causing a proptosis of the right eye ((**c**) black arrow). The optic nerve and extra-ocular muscles were involved by the mass (**b**,**c**). The histologic examination after mass biopsy revealed diffuse nerve fascicles distension caused by a proliferation of spindle-shaped cells embedded in myxoid matrix (hematoxylin-eosin (EE), 2.5×) consistent with plexiform neurofibroma (PN) (**d**).

**Figure 2:**
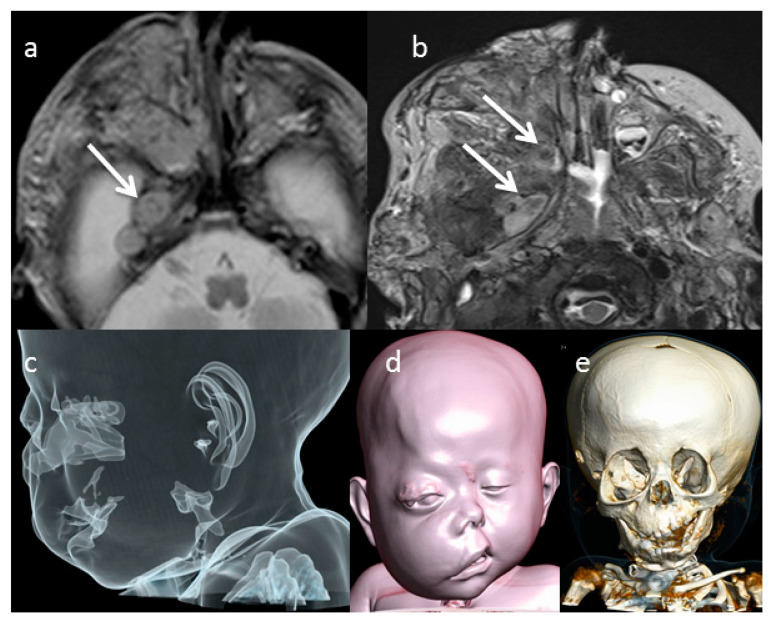
Axial T2-w MRI performed at diagnosis (**a**) and at the follow-up (**b**) showed the typical “target sign” represented by a rim of high signal peripherally and low signal centrally ((**a**,**b**) white arrows). Computed tomography (CT) 3D reconstructions (**c**–**e**) showed the swelling of the cheek and the ocular proptosis of the right eye (**d**) and the orbital enlargement (**e**).

## Data Availability

The data presented in this study are available on request from the corresponding author.
